# Development of Cholesterol-Lowering and Detox Formulations Using Bentonite and Herbal Ingredients

**DOI:** 10.3389/fphar.2021.775789

**Published:** 2021-12-06

**Authors:** Rana Turgut, Murat Kartal, Esra Küpeli Akkol, İlker Demirbolat, Hakkı Taştan

**Affiliations:** ^1^ Department of Pharmacognosy, Health Sciences Institute, Bezmialem Vakif University, Istanbul, Turkey; ^2^ Department of Pharmacognosy, Faculty of Pharmacy, Bezmialem Vakif University, Istanbul, Turkey; ^3^ Department of Pharmacognosy, Faculty of Pharmacy, Gazi University, Ankara, Turkey; ^4^ Bezmialem Center of Education, Practice, and Research in Phytotherapy, Bezmialem Vakif University, Istanbul, Turkey; ^5^ Department of Biology, Faculty of Science, Gazi University, Ankara, Turkey

**Keywords:** detoxification, cholesterol, flaxseed, psyllium, turmeric, grape seed, bentonite

## Abstract

Detoxification enzymes involved in human metabolism works to minimize the potential xenobiotic-induced damage constantly. Studies have revealed that toxin accumulation plays an important role in the etiology of cardiovascular disease. This study has been designed to provide evidence of medicinal use of bentonite, turmeric (*Curcuma longa* L.), grape (*Vitis vinifera* L.) seed, flaxseed (*Linum usitatissimum* L.), and psyllium (*Plantago ovata* L.) as detoxification and cholesterol-lowering agents using a hypercholesterolemic model in mice. The potential hypocholesterolemic effects and detoxification ability of these ingredients were evaluated at the same time: Total cholesterol, high-density lipoprotein cholesterol, low-density lipoprotein cholesterol, triglyceride, glucose, aspartate aminotransferase, alanine aminotransferase, malondialdehyde, plasma total antioxidant activity, nitric acid, leptin levels and glutathione, glutathione peroxidase, lipid peroxidation, superoxide dismutase and catalase values were measured. It was determined that GBTF group (grape seed extract, bentonite, turmeric, and flaxseed), GBTP group (grape seed extract, bentonite, turmeric, and psyllium), and GBT group (grape seed extract, bentonite, and turmeric) of the tested materials decreased the serum total cholesterol concentration by 64.8, 57.5, and 48.9%, respectively, in mice fed a high cholesterol diet. In addition, it was determined that some detoxification parameters such as superoxide dismutase, catalase, glutathione, and glutathione peroxidase were statistically significantly reversed in GBTF, GBTP, and GBT groups. Flaxseed, psyllium, and bentonite clay did not show significant effects in reducing total cholesterol; however, GBTF, GBTP, and GBT groups interventions had a significant effect in reducing total cholesterol levels. Moreover, it was observed that adding flaxseed or psyllium to the GBT group increased the cholesterol-lowering effect. Therefore, it can be thought that this significant effect is due to the synergistic effect of the raw materials. When the results obtained were evaluated, it was seen that the cholesterol-lowering and detoxification effects of the combinations were higher than from the effect of natural material used alone. As a result, combinations of some of these ingredients have a positive effect on reducing the risk of cardiovascular disease.

## 1 Introduction

We are exposed to many xenobiotics during our lifetime, including various pharmaceuticals and food components. Accumulated data suggest that an individual’s ability to remove toxins from the body may play a role in the etiology or exacerbation of a range of chronic conditions and diseases. Detoxification is not one reaction but rather a process that involves multiple reactions and multiple players (Liska, 1998). The liver is the principal organ responsible for detoxifying xenobiotics, including drugs and toxic endogenous compounds. The detoxification process, also known as xenobiotic metabolism, involves three phases with different reactions: phase I (enzymatic functionalization), phase II (enzymatic conjugation), and phase III (transport) (Nakata et al., 2006). Numerous studies have elucidated the enzymes that are crucial in each phase, unraveling the cytochrome P450 (CYPs) in phase I, UDP glycosyltransferases (UGTs), glutathione-S-transferases (GSTs), glutathione peroxidase (GPx), and sulfotransferases (SULTs) in phase II and organic anion transporters (OATs), multidrug-resistance proteins (MDRs) and multidrug resistance-associated proteins (MRPs) in phase III (Chen et al., 2016). Also, to avoid reactive oxygen species (ROS) caused oxidative damage, higher organisms have evolved a complex antioxidant defense system comprising low-molecular-weight components such as ascorbate and glutathione, and enzymatic components such as SOD, peroxidase (POD), and catalase (CAT), which are involved in the detoxification of O_2_ and H_2_O_2_, respectively, thereby preventing the formation of HO radicals (Valko et al., 2007). The detoxification systems are highly complex, show a significant amount of individual variability, and are incredibly responsive to an individual’s environment, lifestyle, and genetic uniqueness (Liska, 1998). In general, the nature of studies indicates that specific foods may upregulate or favorably balance metabolic pathways to assist with toxin biotransformation and subsequent elimination. Therefore, it would seem that designing clinical recommendations to maximize the effects of food and reduce the impact of toxins is essential (Hodges and Minich, 2015).

Cholesterol is the principal sterol present in animal tissues. In mammals, cholesterol plays a vital role in life, being an essential component for the normal functioning of cells. However, cholesterol has gained a bad reputation in the World of health and nutrition, mainly because of its association with cardiovascular diseases. WHO predicted that by the year 2030, CVDs would remain a significant cause of death, affecting approximately 23.6 million people around the World (Cerqueira et al., 2016). Treatment of hypercholesterolemia is currently with a combination of diet and pharmaceutical treatments (Newby et al., 2005; Aarden et al., 2011). Cholesterol-lowering nutraceuticals and functional foods play an essential role in reducing the risk of coronary heart disease by improving the plasma lipoprotein profile (Chen et al., 2008).

Bentonite is a rock formed of highly colloidal and plastic clays composed mainly of montmorillonite, a clay mineral of the smectite group (Adamis et al., 2005). Montmorillonite has an extensive exchange capacity, and thus it can adsorb organic materials, bacteria, viruses, heavy metal ions, and other toxins (Uddin, 2018). In addition, the positive effects of montmorillonite in preventing hyperlipidemia suggest that it may be an excellent nutraceutical to adsorb excess lipids during the consumption of a fatty diet (Xu et al., 2016). Flaxseed’s antioxidant and anti-inflammatory components (*Linum usitatissimum*) are associated with numerous health benefits, including cardiovascular benefits (Chikara et al., 2018). Flaxseed offers the unique opportunity to control different aspects of cardiovascular disease and its risk factors (Parikh and Pierce, 2019). Seeds from *Plantago ovata*, also known as psyllium, have a fiber-rich husk (Belorio and Gómez, 2020). Psyllium has been used traditionally since antiquity as a laxative, but at present, its new pharmacological uses have been discovered (Bannayan et al., 2008). Psyllium husk plays a crucial role in lowering serum cholesterol, so psyllium is considered a potential supportive agent in the therapy of hyperlipidemia (Xing et al., 2017). Turmeric (*Curcuma longa*) is a member of the Zingiberaceae family, and it grows in tropical and subtropical regions around the World (Paramasivam et al., 2009). Its most crucial active ingredient is curcuminoids: Curcumin, dimethoxy curcumin, and bisdemethoxycurcumin (Amalraj et al., 2017). Curcumin can be used as an antioxidant at 1.5% and an antilipidemic agent at 2.5% in the diet (Arshami et al., 2013). Grapes (*Vitis vinifera*) are one of the most highly consumed fruits across the World. In ancient Europe, grape plants’ leaves and sap have been used in traditional treatment for ages. Besides being a wellspring for vitamins and fiber, the skin and seeds of grapes are highly rich in Polyphenols, specifically proanthocyanidins (Gupta et al., 2020).

The main objective of this study was to investigate the effects of potentially effective herbal ingredients and food-grade bentonite clay combinations in detoxifying and normalizing blood cholesterol levels to prevent cardiovascular diseases and develop effective formulations. In this study, bentonite, flaxseed (*Linum usitatissimum*), psyllium (*Plantago ovata*) seed, turmeric (*Curcuma longa*), and grape (*Vitis vinifera*) seed ingredients were determined as potential effective raw materials based on previous researches.

## 2 Materials and Methods

### 2.1 Materials

Food-grade bentonite clay powder was provided by Alya Mineral Company, Ordu, Turkey. Fitovizyon Natural Products Company, Istanbul-Turkey, provided turmeric (*Curcuma longa*) extract. Flaxseed (*Linum usitatissimum*), grape (*Vitis vinifera*) seed, and psyllium (*Plantago ovata*) seed were provided by a local herbalist. Atorvastatin was obtained from the local pharmacy.

### 2.2 Animals

The study’s experiments were performed on mice obtained from the SYLAB Experimental Animal Research and Breeding Laboratory (Ankara, Turkey). This study is a portion of the leading project submitted to comply with the requirements for the degree of Master in Pharmacognosy and Naturel Products Chemistry. This study was supported by the Bezmialem Vakıf University (Istanbul, Turkey) Scientific Research Projects Unit, BAP. Male Swiss albino mice (20–25 g) were used in the study. They were accommodated at the Saki Yenilli Experimental Animal Research and Breeding Laboratory (Ankara, Turkey), where the surrounding temperature was controlled to be between 21 and 24°C for 12 h day and night period. Tap water and standard pellet feed were made freely available to all the animals. According to the National Institutes of Health Guide for Laboratory Animal Care and Use, animal ethical protocols were followed. The animal study was reviewed and approved by Saki Yenilli Experimental Animals Local Ethics Committee (Ethics Committee No: 03/15). At least seven animals were used in each group. All test materials were suspended in 0.5% sodium carboxymethyl cellulose (CMC) and administered to animals by gastric gavage. Two control groups were used in the study. Animals in the hypercholesterolemic group (positive control) were given a high cholesterol diet as in the test samples. The animals in the negative control group were given standard pellet feed without any intervention. Atorvastatin as a reference substance was administered orally to animals by gastric gavage at a dose of 10 mg/kg.

### 2.3 Analysis of Fixed Oils From Grape Seeds, Flaxseed, and Psyllium Seed

The seeds were powdered mechanically and extracted with hexane (180°C) for 5–6 h in a Soxhlet apparatus (Elektromag, Turkey). Removal of the solvent under reduced pressure gave the fixed oils. The fatty acid content of the fixed oils was investigated by GC analysis of their methyl esters. İnto the oils (0.5 ml) 5 ml 10% methanolic HCl was added. Methyl esters were prepared by transmethylation at 180°C 30 min heating under reflux. 5 ml of hexane, 25 ml of 10% NaHCO_3_, and salt were added to the mixture, respectively, and shaken vigorously, and allowed to stand for 30 min. The upper layer was removed, and 1 μL was used for GC analysis (Houghton et al., 1995).

Gas Chromatography-Mass Spectrometry (GS-MS) is used to identify the fatty acid components of fixed oils, and Gas Chromatography Flame ionization Detector (GC-FID) is used to determine relative percentages. GC-FID analyses were performed using an Agilent 7890B Gas Chromatograph system with an Agilent DB-Wax capillary column (60 m × 0.25 mm × 0.25 µm) and helium as the carrier gas. Injector and detector (FID) temperatures were kept at 220°C. The split ratio was 50:1. Relative percentage amounts were calculated from the total area under the peaks by the software of the apparatus. GC–MS analyses of the oils were carried out on an Agilent 75977E Gas Chromatograph system. The oven temperature was as above; transfer line temperature 250°C; ion source temperature 230°C; carrier gas helium; ionization energy 70 eV; mass range 35–450 m/z.

### 2.4 Analysis of Swelling Index of Flaxseed and Psyllium Seed

The swelling index is the volume in ml taken up by the swelling of 1 g of plant material under specified conditions. For the characterization of mucilage content, the swelling index was determined according to the general and specified descriptions of European Pharmacopoeia by using *Linum usitatissimum* and *Plantago ovata* 1 g whole and powder seeds at the same time.

### 2.5 Preparation of Grape Seed Extract

The seeds were extracted for 5–6 h in a Soxhlet apparatus (Electromag, Turkey) with ethyl alcohol at different ratios (100% ethyl alcohol, 70% ethyl alcohol/water, and 50% ethyl alcohol/water) by temperature controlled. Evaporation was carried out at different temperatures for each solvent. As a result of the total phenolic/flavonoid content analysis, the appropriate solvent was determined as 50% ethanol/water. The above method was applied for the grape seed extract to be used in the study.

### 2.6 Analysis of Total Phenolic and Total Flavanoid Contents of Grape Seed Extract

Two different colorimetric methods determined the total phenolic and flavonoid content. The total phenolic content of the extract was determined by the Folin Ciocalteu reagent (FCR). UV-VIS Spectrophotometry device was used for the Folin Ciocalteu Method. After necessary dilution for the sample, the test solution was prepared with 104 µL of distilled water, 8 µL of the sample, 8 µL of FCR, and 80 µL of 7% Na_2_CO_3_. The test solution was kept in the dark for 90 min. Then, absorbance at 765 nm was determined against a blank containing all reagents, without the samples or the gallic acid. The total phenolic content is the number of gallic acid equivalents.

The total flavonoid content of the extract was determined by the aluminum chloride method, using quercetin as a reference compound. UV-VIS Spectrophotometry device was used for Aluminum Chloride Colorimetric Method. After necessary dilution for the sample, the test solution was prepared with 134 µL of distilled water, 20 µL of the sample, 6 µL of 10% AlCl3, and 40 µL of CH3COONH4. The test solution was kept in the dark for 10 min. Then, the same absorbance at 765 nm was determined under the same conditions. The total flavonoid content is the number of quercetin equivalents.

### 2.7 LC-HRMS Evaluation

Liquid Chromatography-High Resolution Mass Spectrometer (LC-HRMS) device was used for phenolic compound determination in grape seed extract. The extract was dissolved in MeOH-water, and a final concentration of 3 ppm was added from the 100 mg/L internal standard solutions. The sample was passed through a 0.45 μ filter, and 2 μ was added to the device. Separation was done on the C18 guard column and the mobile phase used was formic acid: water: Methanol. The rate of flow was constantly maintained at 0.35 ml per minute, and the peaks that appeared were recognized with the help of the mass scanning range 100–900 m/z. An MS detector was used for the segregation of phenolic compounds. The identification of these phenolic compounds was done by comparing the retention time and the spectral peaks previously obtained by the injection of standards. The calculation for their quantification was performed by external calibration.

### 2.8 Analysis of Curcumin in Turmeric Extract

10 mg of turmeric extract was dissolved in 10 ml of Methanol by staying in an ultrasonic bath for 10 min. 100 μL of the prepared solution was taken and diluted into 10 ml of mobile phase mixture. Curcumin amount was measured by HPLC-PDA Method (standard substance Curcumin; Sigma Aldrich).

### 2.9 Determination of Intervention Doses

Food-grade bentonite clay powder was determined as 60 mg/day for each mouse (Gershkovich et al., 2009; EFSA Panel on Additives and Products or Substances used in Animal Feed (FEEDAP), 2012). Flaxseeds (*Linum usitatissimum*) were determined as 7 mg/day for each mouse (Pan et al., 2009). Grape (*Vitis vinifera*) seed extract was determined as 2.5 mg/day for each mouse (EFSA Panel on Additives and Products or Substances used in Animal Feed (FEEDAP), 2016). Turmeric (*Curcuma longa*) extract was determined as 2 mg/day for each mouse (Qinna et al., 2012). Psyllium (*Plantago ovata*) was determined as 7 mg/day for each mouse (Wei et al., 2009).

### 2.10 Groups

When the mean difference was 4.77 units and the standard deviation was 3.52, the sample size was determined as six experimental animals for each group, a total of n = 54 (95% confidence level and 80% power at α = 0.05 significance level). The 54 adult male Swiss albino mice were divided into nine groups (*n* = 6) are shown in Table 1.

**TABLE 1:** Study groups.

Groups	Intervention
1. Control group	Only standard diet
2. Hypercholesterolemic group	Only high cholesterol diet (HCD)
3. Reference group	HCD + Atorvastatin (10 mg/kg/day)
4. Bentonite group	HCD + bentonite clay (60 mg/day)
5. GBTF group	HCD + bentonite clay (60 mg/day) + flaxseed (7 mg/day) + grape seed extract (2.5 mg/day) + turmeric extract (2 mg/day)
6. GBTP group	HCD + bentonite clay (60 mg/day) + psyllium (7 mg/day) + grape seed extract (2.5 mg/day) + turmeric extract (2 mg/day)
7. GBT group	HCD + bentonite clay (60 mg/day) + grape seed extract (2.5 mg/day) + turmeric extract (2 mg/day)
8. Flaxseed group	HCD + flaxseed (7 mg/day)
9. Psyllium group	HCD + psyllium (7 mg/day)

Grape seed extract contains 24.81% palmitic acid, 6.02% palmitoleic acid, 6.10% stearic acid, 15.26% oleic acid, 43.61% linoleic acid, 4.20% linolenic acid, 24.6% phenolic substance and 1.48% flavonoid substance, flaxseed contains 9.27% palmitic acid, 5.9% stearic acid, 22.61% oleic acid, 17.83% linoleic acid, 44.27% linolenic acid and the swelling index 4.7 ml for ground flaxseed, psyllium contains 15.25% palmitic acid, 4.18% stearic acid, 25.96% oleic acid, 48.42% linoleic acid, 6.17% linolenic acid and the swelling index 17.8 ml for ground psyllium seed and turmeric extract contains 95% curcumin from the products used in this study.

### 2.11 Hypercholesterolemia Experimental Design

Hypercholesterolemia was induced by the applying pellet feed containing 1% cholesterol to the experimental animals for 30 days. The test materials were administered simultaneously to the mice in the test group (except the control group). At the end of the 30-days experiment period, intracardiac blood samples and liver tissue were taken from the mice in all groups for biochemical determinations. At the end of the period, blood and tissue samples were taken from experimental animals;

1) Total cholesterol (TC), HDL cholesterol (HDL-C), LDL cholesterol (LDL-C) and Triglyceride (TG) values
2) Glucose, Aspartate aminotransferase (AST), Alanine aminotransferase (ALT), Malondialdehyde (MDA), Plasma total antioxidant activity (TAA), Nitric acid levels
3) Glutathione (GSH), Glutathione peroxidase (GPx), Lipid peroxidation (LPO), Superoxide dismutase (SOD), Catalase (CAT) values
4) Leptin levels were measured.

### 2.12 Estimation of Lipid Peroxidation Level in Serum

The method (Kurtel et al., 1992) was used for the measurement of lipid peroxidation levels in serum. In this method, 1 ml of serum, 2 ml of trichloroacetic acid (TCA; 15%)-thiobarbituric acid (TBA; 0.375%), 0.25 N HCl was mixed and centrifuged at 10,000 g for 5 min. After the supernatant was separated, it was mixed with 20 µL of butylhydroxytoluene (BHT) to prevent oxidation and kept in a hot water bath for 15 min. After cooling under running water, the precipitate was separated by centrifugation at 10,000 g for 5 min. The absorbance of the sample was then measured at 532 nm.

### 2.13 Determination of Antioxidant Parameters in Liver Tissue

The reduced glutathione (GSH) parameter, which is a non-enzymatic antioxidant, was determined by the method of Sedlak and Lindsay (Sedlak and Lindsay, 1968). With the method of Paglia and Valentine (Paglia and Valentine, 1967), the GPx parameter was determined. Enzymatic antioxidant superoxide dismutase (SOD) activity by the method developed by Misra and Fridovich (Misra and Fridovich, 1972) was determined. A catalase (CAT) activity test with the method determined by Takahara et al. (1960) was done. The lipid peroxide (LPO) content in tissues was determined according to the method of Ohkawa et al. (1979).

### 2.14 Determination of Other Parameters

Serum high-density lipoprotein cholesterol (HDL-C) levels were determined using the Crescent Diagnostics Cholesterol Test Kit by precipitation of apolipoprotein B-containing lipoproteins with phosphotungstic acid and magnesium chloride. The cholesterol content of low-density lipoprotein (LDL-C) was determined using the Friedwald equation. Alanine aminotransferase and aspartate aminotransferase levels were determined using commercial kits from the Teco Diagnostics assay. Serum total protein, triglyceride, and cholesterol values were measured by enzymatic methods using commercially available kits (TECO Diagnostics, California, United States). Malondialdehyde was measured according to the method of Draper and Hardley (Draper and Hadley, 1990) based on the binding of MDA with thiobarbituric acid. The standard Fe-EDTA complex solution reacts with hydrogen peroxide in a Fenton-type reaction, leading to hydroxyl radicals (•OH). These reactive oxygen species break down the benzoate, causing the release of TBARS. Antioxidants cause suppression of TBARS production. The plasma total antioxidant activity test, which is based on the spectrophotometric measurement of the inhibition of color formation, defined as TAA, was measured according to the method of Koracevic et al. (2001). Nitric oxide metabolites (nitrates + nitrites, NOx) were tested in plasma by the colorimetric method of Griess (Miranda et al., 2001). Plasma leptin concentration was determined by the ELISA (Linco Research, Inc, St. Charles, United States) method using the enzyme kit (Cat. EZRL-83K).

### 2.15 Histopathological Analysis

The liver tissue of rats was dissected and then placed in formalin (10%) for 3 days. All tissues were detected using the Thermo Scientific Excelsior (ES) machine. The tissues were embedded in paraffin wax and blocks were prepared using the HistoCentre 2 machine. Subsequently, sections of 3.5 mm thickness were made from paraffin-embedded blocks using a Leica RM2255 microtome. The sections were stained with hematoxyline-eosin (HE) using the Shandon Varistan machine. Photographs of normal and pathological liver tissues were taken using Nikon Eclipse Ci with polarizing attachment and a Digital Image analysis system, then examined under a light microscope.

### 2.16 Gastric Ulcerogenic Effect

Potential risks for stomach damage were assessed due to the application of test samples over a long period, such as 30 days. For this purpose, after the hypocholesterolemic activity test was completed, the mice were killed using a high-dose anesthetic, and their stomachs were removed. It was examined under a dissecting microscope to determine the lesions or bleeding that may develop in the stomach.

### 2.17 Statistical Analysis

Experiment results were expressed as Mean Standard Error ± SEM. The effect of test samples on the parameters studied was compared with the results obtained in the hypercholesterolemic group, and statistical differences were evaluated by ANOVA and Student-Newman-Keuls posthoc tests. The difference in *p* < 0.05 was considered significant (*p < 0.05; **p < 0.01; ***p < 0.001).

### 2.18 Exclusion Criteria

1) Behavioral changes, goose pimples, hunched position, vomiting, diarrhea, formation of secondary skin lesions
2) Loss (ex) of animals during the experiment

No animals included in the study were excluded from the experiment, as no negative findings were found among the criteria mentioned above.

## 3 Results

### 3.1 Gas Chromatography Analysis

**FIGURE 1:**
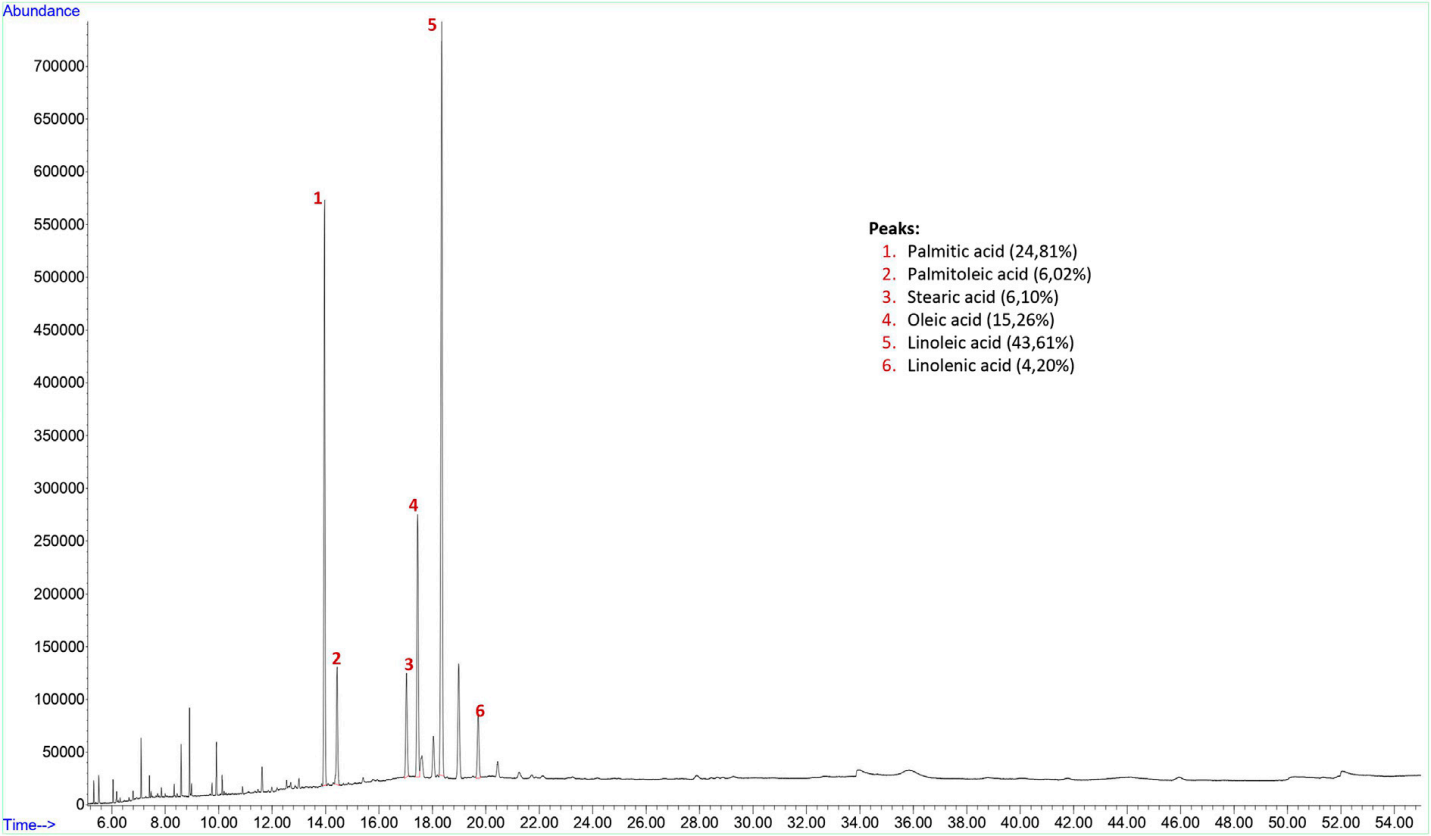
GC-FID chromatogram of hexane extract of grape (*Vitis vinifera*) seed.

The yield of the hexane extract obtained for the analysis of grape (*Vitis vinifera*) seed fixed oil was 9.4%. Findings of fixed oil acid components and relative percentages of fixed oils in grape seed are shown in Figure 1. The GC-FID chromatogram of the hexane extract of the grape seed found palmitic acid (24.81% **±** 2.50), palmitoleic acid (6.02% **±** 2.08), stearic acid (6.10% **±** 1.22), oleic acid (15.26% **±** 1.77), linoleic acid (43.61% **±** 4.25), and linolenic acid (4.20% **±** 0.94) present in the seed as fixed oil acid constituents (Figure 1).

**FIGURE 2:**
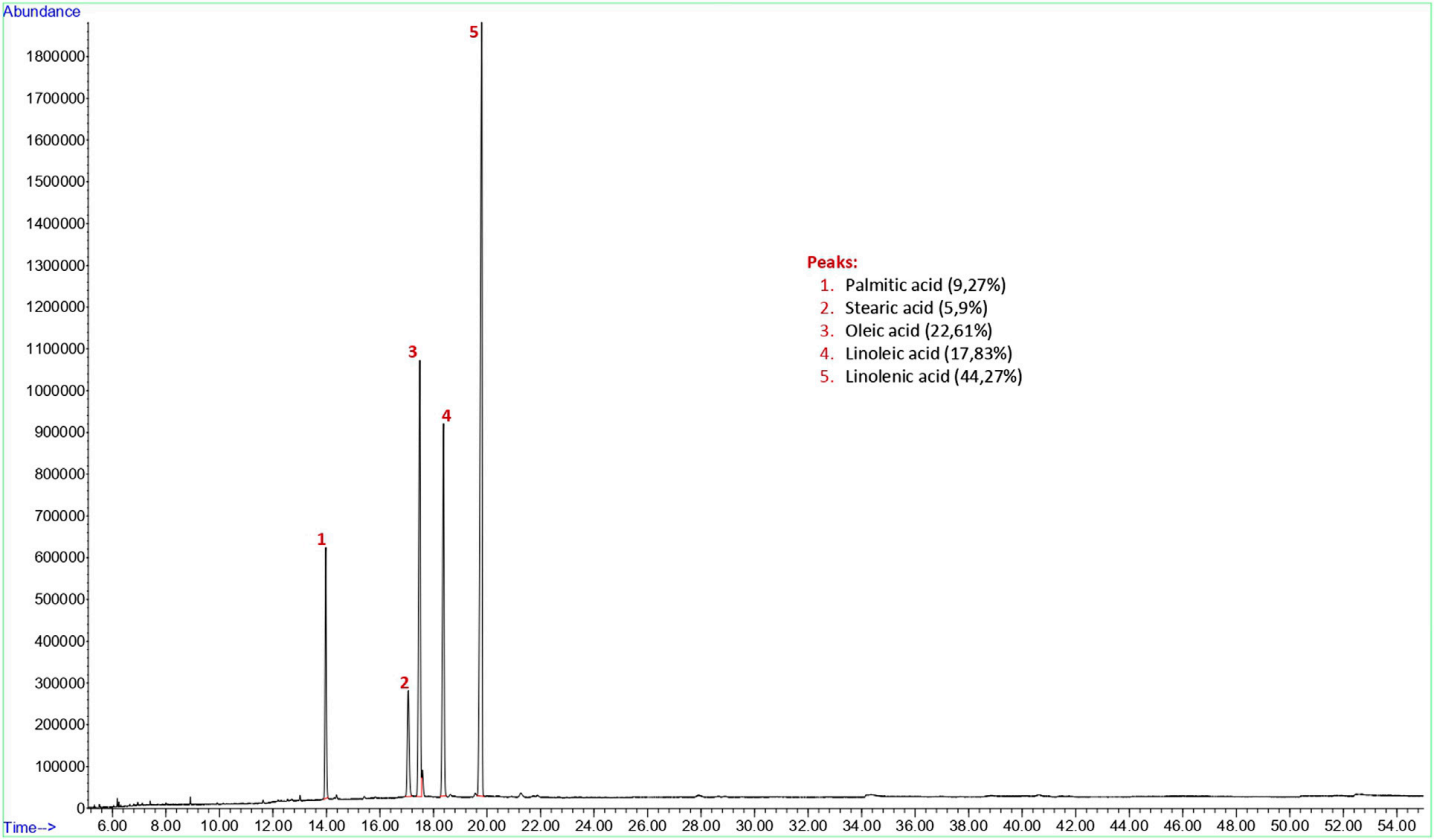
GC-FID chromatogram of hexane extract of flax (*Linum usitatissimum*) seed.

The yield of the hexane extract obtained for the analysis of flax (*Linum usitatissimum*) seed fixed oil was 15.3%. Findings of fixed oil acid components and relative percentages of fixed oils in flaxseed are shown in Figure 2. The GC-FID chromatogram of the hexane extract of the flaxseed found palmitic acid (9.27% **±** 0.62), stearic acid (5.9% **±** 1.40), oleic acid (22.61% **±** 2.14), linoleic acid (17.83% **±** 0.47) and linolenic acid (44.27% **±** 2.21) present in the seed as fixed oil acid constituents (Figure 2).

**FIGURE 3:**
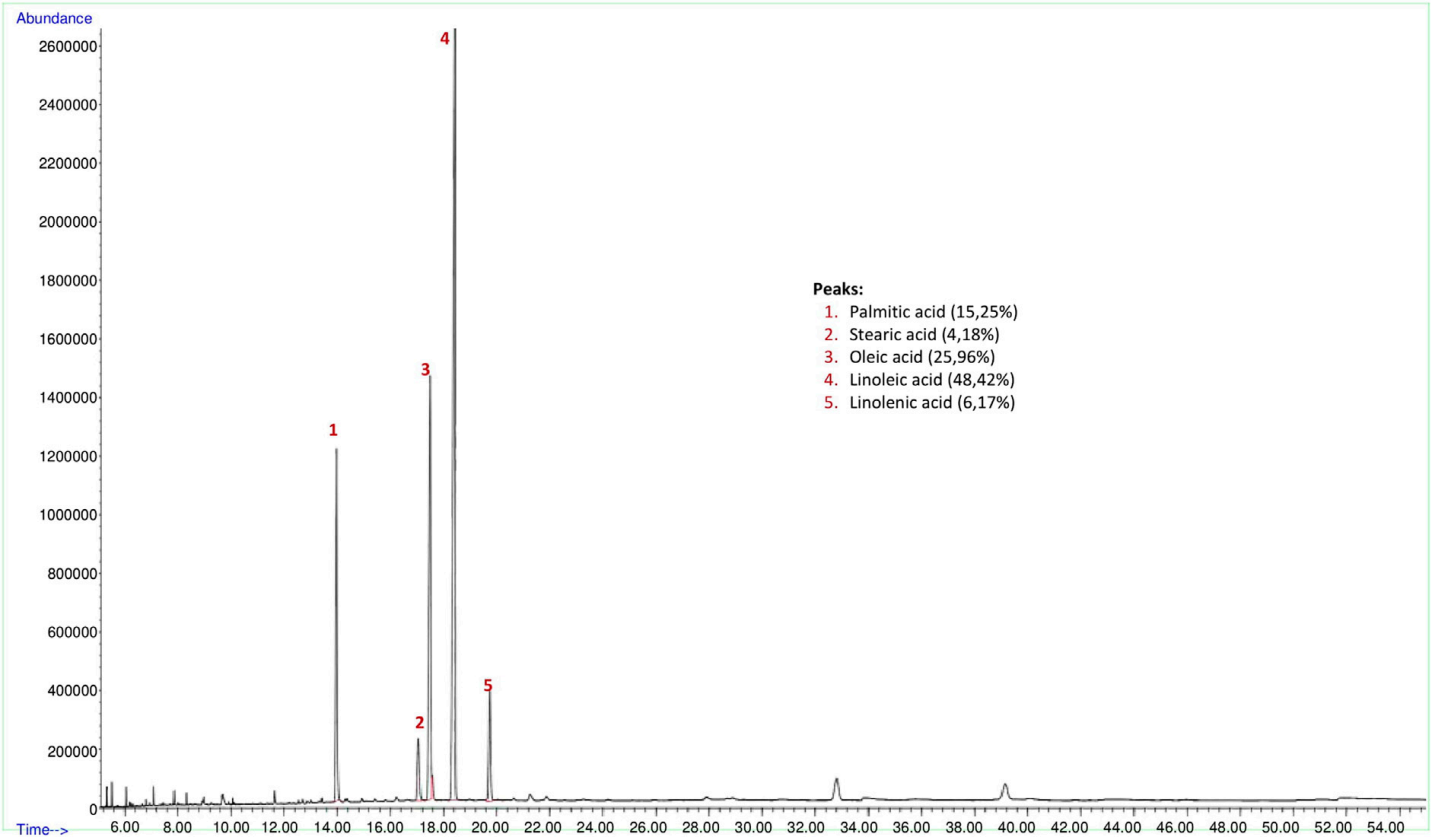
GC-FID chromatogram of hexane extract of psyllium (*Plantago ovata*) seed.

The yield of the hexane extract obtained for the analysis of psyllium (*Plantago ovata*) seed fixed oil was 2.3%. Findings of fixed oil acid components and relative percentages of fixed oils in psyllium seed are shown in Figure 3. The GC-FID chromatogram of the hexane extract of the psyllium seed found palmitic acid (15.25% **±** 0.94), stearic acid (4.18% **±** 1.05), oleic acid (25.96% **±** 3.06), linoleic acid (48.42% **±** 2.70) and linolenic acid (6.17% **±** 0.21) present in the seed as fixed oil acid constituents (Figure 3).

### 3.2 Findings of the Swelling Index

It is stated in the European Pharmacopoeia that the swelling index for flaxseed should be at least 4 ml. According to the average of three parallel experiments, the swelling index was found to be 3.8 ml (±0.1) in whole flaxseed and 4.7 ml (±0.17) in-ground flaxseed.

It is stated in the European Pharmacopoeia that the swelling index should be at least 10 ml for psyllium seeds. According to the average of three experiments performed in parallel, the swelling index was found to be 9.3 ml (±0.56) in the whole psyllium seed and 17.8 ml (±0.45) in the ground psyllium seed.

### 3.3 Total Phenolic and Total Flavonoid Contents of Grape Seed Extract

According to the analysis of total phenolic and flavonoid content in grape seed extracts obtained using different solvents (100% ethyl alcohol, 70% ethyl alcohol/water, and 50% ethyl alcohol/water), the final solvent was determined as 50% ethyl alcohol/water.

In the grape seed extracts obtained by using the final solvent, the phenolic substance content was 24.6% **±** 5.57 and the flavonoid substance amount was 1.48% **±** 0.38 in the average of the results of the three-repeated analysis.

### 3.4 Liquid Chromatography-High Resolution Mass Spectrometer Analysis

The analysis results of the determination of the number of phenolic substances in grape (*Vitis vinifera*) seed extract are shown in Table 2. According to the analysis results, the phenolic compounds in the grape seed extract were chlorogenic acid, fumaric acid, (-)-epicatechin gallate, caffeic acid, vanillic acid, luteolin 7-glucoside, resveratrol, apigenin 7-glucoside, quercetin, luteolin, and apigenin.

**TABLE 2:** Determined phenolic percentage components of grape seed extract.

Phenolic compounds	%
(-)-Epicatechin gallate	3.05
Apigenin	2.87
Apigenin 7-glucoside	3.59
Caffeic acid	3.74
Chlorogenic acid	3.58
Fumaric acid	2.88
Luteolin	3.42
Luteolin 7-glucoside	4.14
Quercetin	2.95
Resveratrol	2.87
Vanillic acid	3.49

Grape seed extract used in this study contains 24.6% phenolic substance and 1.48% flavonoid substance. These phenolic substances are chlorogenic acid, fumaric acid, (-)-epicatechin gallate, caffeic acid, vanillic acid, luteolin 7-glucoside, resveratrol, apigenin 7-glucoside, quercetin, luteolin, and apigenin as you can see Table 2.

### 3.5 High-Performance Liquid Chromatographic Analysis

**FIGURE 4:**
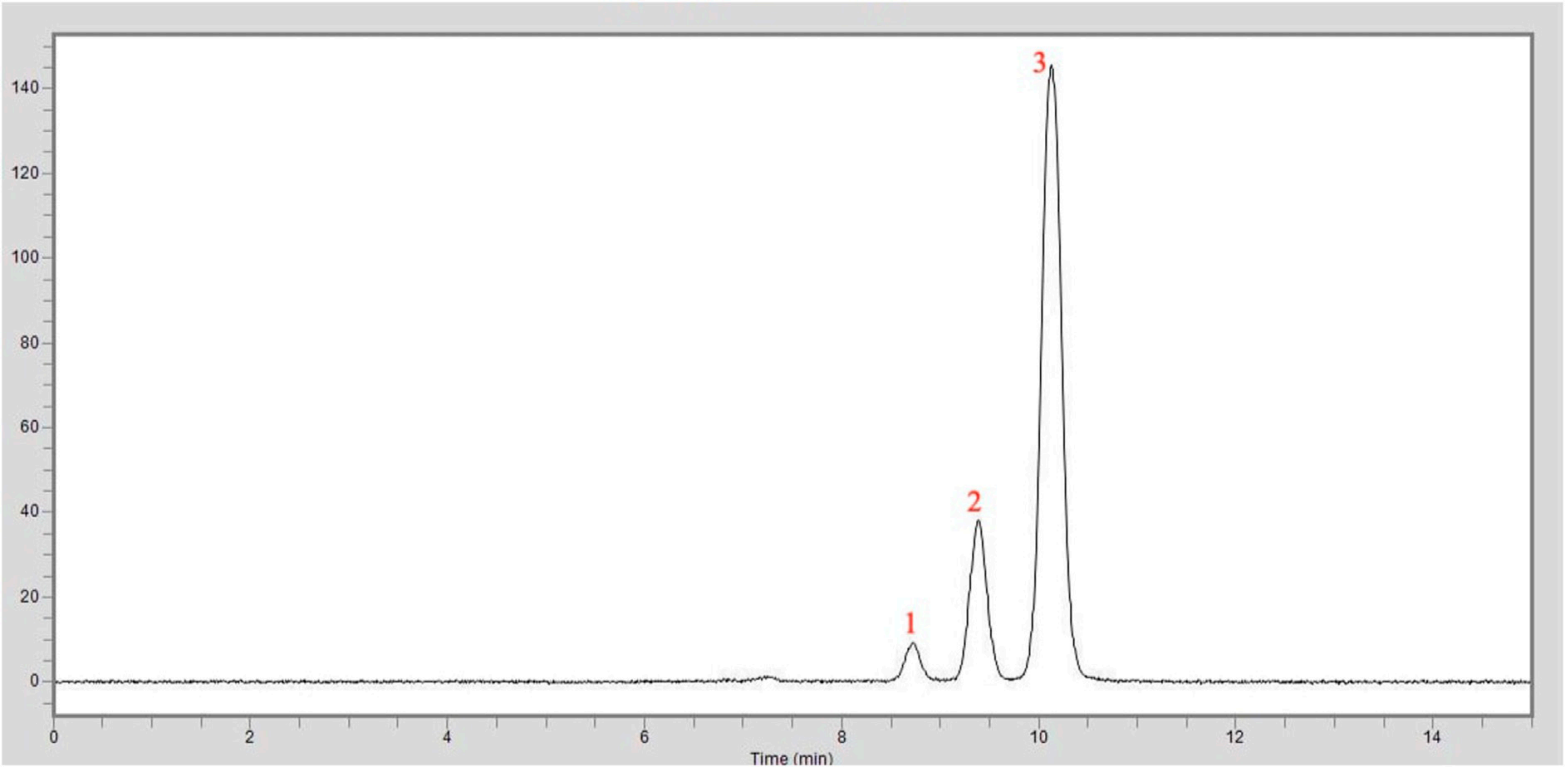
HPLC chromatogram of the curcumin analysis in the turmeric extract: 1. Peak: Bisdemethoxycurcumin, 2. Peak: Demethoxycurcumin, 3. Peak: Curcumin.

The HPLC chromatogram of the curcumin analysis result of the turmeric extract used in this study is shown in Figure 4. The sample was tested under the same conditions with the standard curcumin substance, and the amounts of curcumin were calculated from the peak areas in the same retention time. As a result, it was shown that the turmeric extract used in this study contains 95% curcumin.

### 3.6 Effect of the Interventions on Serum Biochemical Parameters

Our study determined that mice fed a high cholesterol diet had a higher serum total cholesterol concentration than mice fed a regular diet. The effects of test materials on serum lipid profile are shown in Table 3. Of the test materials, GBTF Group, GBTP Group, and GBT Group were found to reduce serum total cholesterol concentration by 64.8, 57.5, and 48.9%, respectively, without causing any ulcerogenic effect on the stomach surface in mice fed with a high cholesterol diet. Total cholesterol levels in blood samples obtained from GBTF Group and GBTP Group were significantly lower compared to the hypercholesterolemic group (*p* < 0.01). Although the total cholesterol levels obtained from the GBT Group were significant compared to the hypercholesterolemic group, the significance level was lower than GBTF Group and GBTP Group (*p* < 0.05). As shown in Table 3, all studied test samples showed increased serum HDL-C level activity, but this sign was higher in GBTF Group, GBTP Group, GBT Group, and Flaxseed Group. HDL-C levels were significantly higher in blood samples obtained from GBTP Group than the hypercholesterolemic group (*p* < 0.01). Although HDL-C levels obtained from GBTF Group, GBT Group, and Flaxseed Group were significant compared to the hypercholesterolemic group, the significance level was lower than GBTP Group (*p* < 0.05).

**TABLE 3:** Effects of test materials on serum TC, HDL-C, LDL-C, and TG levels.

Material	Total cholesterol (mg/dl) (%)	HDL-C (mg/dl) (%)	LDL-C (mg/dl) (%)	Triglyceride (mg/dl) (%)
Control group	127.3 ± 11.6	24.7 ± 1.5	80.7 ± 16.9	116.9 ± 21.3
Hypercholesterolemic group	202.8 ± 14.7^#^	20.1 ± 1.2	153.6 ± 21.5	171.4 ± 18.1
Bentonite group	151.2 ± 19.3 (−25.4)	26.7 ± 1.5 (+24.7)	172.6 ± 20.3 (+12.4)	193.6 ± 21.9 (+12.9)
GBTF group	71.4 ± 9.5** (−64.8)	34.0 ± 1.7* (+40.8)	58.2 ± 17.1** (−62.1)	58.1 ± 12.7** (−66.1)
GBTP group	86.1 ± 7.9** (−57.5)	39.4 ± 1.7** (+48.9)	60.3 ± 6.2** (−60.7)	63.9 ± 14.1** (−62.7)
GBT group	103.6 ± 11.8* (−48.9)	30.6 ± 2.4* (+34.3)	81.7 ± 12.9* (−46.8)	91.7 ± 9.4* (−46.5)
Flaxseed group	182.1 ± 23.4 (−10.2)	29.1 ± 2.2* (+30.9)	119.5 ± 13.7 (−22.2)	103.1 ± 9.7 (−39.8)
Psyllium group	173.1 ± 12.5 (−14.6)	23.4 ± 1.9 (+14.1)	159.1 ± 17.3 (+3.6)	118.2 ± 13.5 (−31.0)
*Atorvastatin (10 mg/kg)*	98.5 ± 12.7* (−51.4)	40.3 ± 1.1** (+50.1)	68.2 ± 13.5** (−55.6)	79.2 ± 11.0* (−53.8)

*p < 0.05; **p < 0.01; ***p < 0.001 (significance relative to hypercholesterolemic group); #: *p* < 0.05 (significance relative to control group).

Bentonite Group: HCD + bentonite clay (60 mg/day); GBTF group: HCD + bentonite clay (60 mg/day) + flaxseed (7 mg/day) + grape seed extract (2.5 mg/day) + turmeric extract (2 mg/day); GBTP group: HCD + bentonite clay (60 mg/day) + psyllium (7 mg/day) + grape seed extract (2.5 mg/day) + turmeric extract (2 mg/day); GBT group: HCD + bentonite clay (60 mg/day) + grape seed extract (2.5 mg/day) + turmeric extract (2 mg/day); Flaxseed Group: HCD + flaxseed (7 mg/day); Psyllium Group: HCD + psyllium (7 mg/day).

It was determined that serum LDL-C and triglyceride levels were significantly reduced in GBTF Group, GBTP Group, and GBT Group. LDL-C levels were found to be significantly lower in blood samples obtained from GBTF Group and GBTP Group compared to the hypercholesterolemic group (*p* < 0.01). Although the LDL-C levels obtained from the GBT Group were significant compared to the hypercholesterolemic group, the level of significance was lower than GBTF Group and GBTP Group (*p* < 0.05). Triglyceride levels were significantly lower in blood samples obtained from GBTF Group and GBTP Group compared to the hypercholesterolemic group (*p* < 0.01). Although the triglyceride levels obtained from the GBT Group were significant compared to the hypercholesterolemic group, the significance level was lower than GBTF Group and GBTP Group (*p* < 0.05).

The effects of test materials on serum glucose, AST, ALT, MDA, TAA, nitric oxide, and leptin levels are shown in Table 4. Our study observed that none of the test materials caused a statistically significant change in serum glucose levels, but the inhibitory ratios in GBTF Group and GBTP Group were remarkable. Remarkable inhibitory effects on serum AST and ALT levels of hepatic marker enzymes were observed in almost all test materials. It was determined that the statistical significance in antihepatotoxic potential was especially in GBTF Group, GBTP Group and GBT Group. In addition, it was determined that the MDA concentration decreased significantly in GBTF Group, and GBTP Group. It was observed that the TAA decreased in the cholesterol-rich diet group compared to the control group. However, there was no statistically significant effect in modulating the decreased plasma TAA level, with a general tendency to normalize the TAA level.

**TABLE 4:** Effects of test materials on serum Glucose, AST, ALT, MDA, TAA, Nitric oxide, and Leptin.

Material	Glucose (mg/dl) (%)	AST (IU/L) (%)	ALT (IU/L) (%)	MDA (nmol/ml) (%)	TAA (mmol/L) (%)	Nitric oxide (µmol/L) (%)	Leptin (µg/L) (%)
Control Group	89.3 ± 13.3	51.4 ± 14.9	11.5 ± 4.9	1.6 ± 0.9	1.22 ± 0.5	21.5 ± 4.3	2.11 ± 0.3
Hypercholesterolomic Group	67.1 ± 8.4	71.5 ± 10.6	39.3 ± 11.6	3.9 ± 1.7	0.94 ± 0.8	25.7 ± 5.8	8.26 ± 1.9
Bentonite Group	52.6 ± 14.1 (−21.6)	69.2 ± 14.7 (−3.2)	31.2 ± 12.4 (−20.6)	3.6 ± 1.1 (−7.7)	0.97 ± 0.3 (+3.2)	27.3 ± 3.2 (+6.2)	7.01 ± 0.7 (−15.1)
GBTF Group	87.5 ± 9.6 (+30.4)	18.4 ± 5.7** (−74.3)	6.4 ± 5.0** (−83.7)	1.3 ± 0.2** (−66.7)	1.13 ± 0.5 (+20.2)	33.8 ± 3.1 (+31.5)	4.82 ± 1.4* (−41.6)
GBTP Group	96.4 ± 11.8 (+43.7)	23.7 ± 9.6** (−66.9)	8.7 ± 9.3** (−77.8)	2.0 ± 0.3* (−48.7)	1.25 ± 0.3 (+32.9)	34.1 ± 7.4 (+32.7)	5.27 ± 1.2* (−36.2)
GBT Group	74.2 ± 12.4 (+10.6)	41.5 ± 13.2* (−41.9)	10.5 ± 4.1** (−73.3)	2.1 ± 0.6 (−46.2)	0.51 ± 0.6 (−45.7)	31.9 ± 4.0 (+24.1)	6.13 ± 1.1 (−25.8)
Flaxseed Group	48.1 ± 9.3 (−28.3)	47.1 ± 11.3 (−34.1)	13.7 ± 9.2* (−65.1)	2.3 ± 0.5 (−41.0)	1.05 ± 0.1 (+11.7)	31.4 ± 6.8 (+22.2)	6.28 ± 0.8 (−23.9)
Psyllium Group	44.6 ± 7.5 (−33.5)	61.4 ± 12.5 (−14.1)	21.1 ± 7.5 (−46.3)	2.5 ± 0.4 (−35.9)	1.09 ± 0.3 (+15.9)	29.6 ± 4.1 (+15.2)	6.94 ± 0.9 (−15.9)
*Atorvastatin (10 mg/kg)*	93.4 ± 8.3 (+39.2)	26.2 ± 9.1** (−63.4)	7.9 ± 8.6** (−79.9)	2.1 ± 0.4* (−46.2)	1.17 ± 0.2 (+24.4)	37.2 ± 4.9 (+44.7)	3.92 ± 1.0** (52.5)

*p < 0.05; **p < 0.01; ***p < 0.001 (significance relative to hypercholesterolemic group).

Bentonite Group: HCD + bentonite clay (60 mg/day); GBTF group: HCD + bentonite clay (60 mg/day) + flaxseed (7 mg/day) + grape seed extract (2.5 mg/day) + turmeric extract (2 mg/day); GBTP group: HCD + bentonite clay (60 mg/day) + psyllium (7 mg/day) + grape seed extract (2.5 mg/day) + turmeric extract (2 mg/day); GBT group: HCD + bentonite clay (60 mg/day) + grape seed extract (2.5 mg/day) + turmeric extract (2 mg/day); Flaxseed Group: HCD + flaxseed (7 mg/day); Psyllium Group: HCD + psyllium (7 mg/day).

Although the hypercholesterolemic diet caused a slight decrease in the plasma concentration of NO metabolites, the test samples showed significant (not statistically significant) increased activity. This study observed a significant increase in body weight and plasma leptin levels in the high-fat diet group compared to the control group animals. GBTF Group and GBTP Group interventions showed a 41.6 and 36.2% inhibition ratio in leptin concentration, respectively.

The lipid peroxidation level in the hypercholesterolemic group was relatively higher than in the control group. However, it was determined that statistically significant inhibitory activity on lipid peroxidation was shown in GBTF Group, GBTP Group, and GBT Group. Our study examined SOD, catalase, GSH, and GPx levels to evaluate oxidative damage in liver tissue. It was shown that all these biochemical parameters were statistically significantly reduced in GBTF Group, GBTP Group, and GBT Group.

**FIGURE 5:**
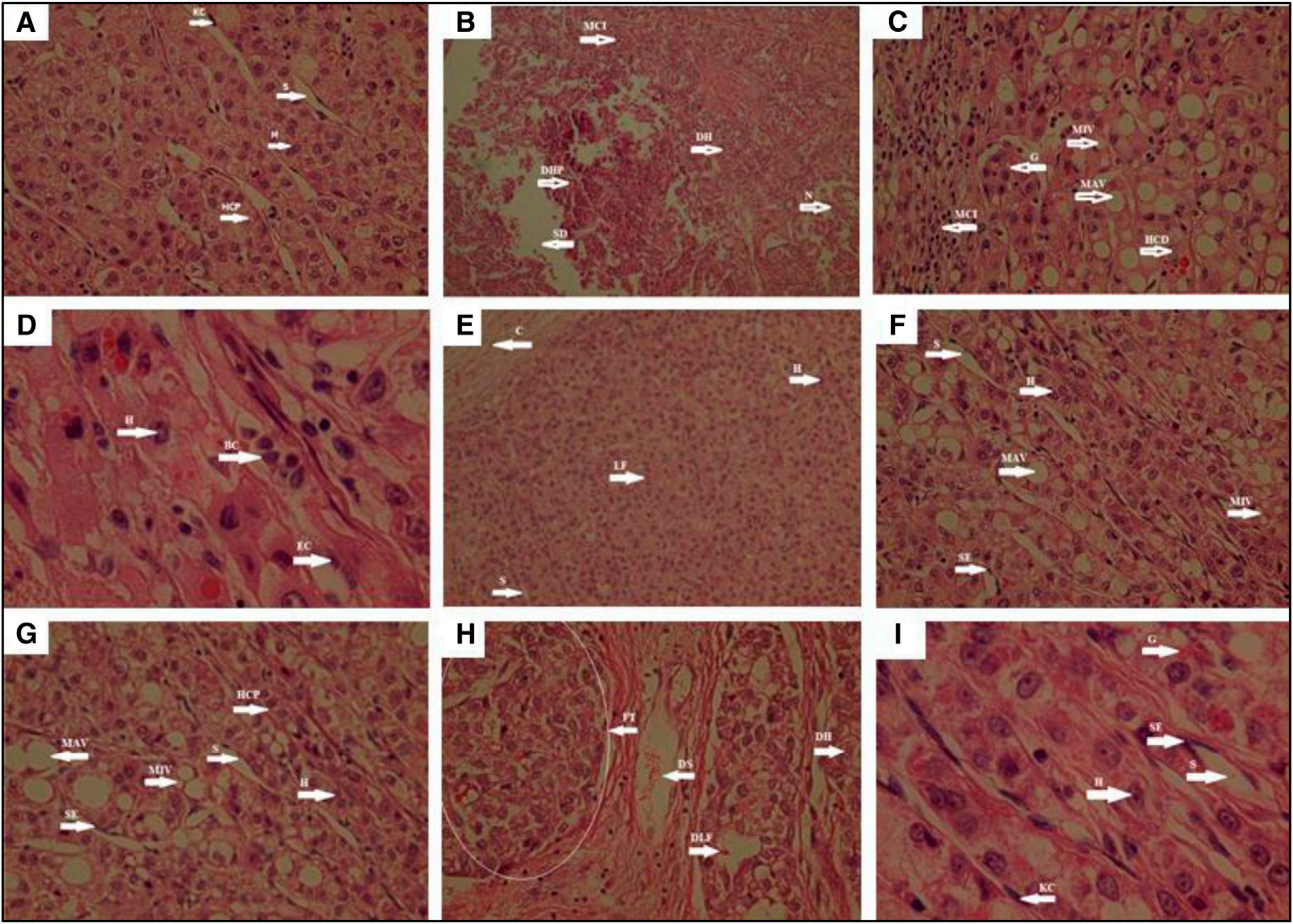
Histopathological view of hepatic tissue in the control, hypercholesterolemia, test materials and reference drug Atorvastatin administered animals. Tissue sections was stained the hematoxylin and eosin (HE). The original magnification was ×100. Data are representative of six animal per group **(A)**: Control group; **(B)**: Hypercholesterolemic group; **(C)**: Bentonite Group; **(D)**: GBTF Group; **(E)**: GBTP Group; **(F)**: GBT Group; **(G)**: Flaxseed Group; **(H)**: Psyllium Group; **(I)**: Atorvastatin. BC, Blood cell in sinusoid; C, Capsule; DH, Degenerative hepatocyte; DHP, Degenerative Hepatocyte plague; DLF, Degenerative lobular formation; DS, Degenerative sinusoid; E, Endothelium; EC, Endothelial cell; FT, Fibrous tissue; G, Granuloma; H, Hepatocyte; HCD, Hepatocyte cell degeneration; HCP, Hepatocyte cell plague; KC, Kupffer cell; LF, Lobular formation; MCI, Mononuclear cell infiltration; MAV, Macrovesicle; MIV, Microvesicle; N, Necrosis; S, Sinusoid; SD, Sinusoid degeneration; SE, Sinusoid endothelium.

According to histopathological analyses, it was determined that there was no degeneration in hepatocytes, hepatocyte cell plague, Kupffer cell, and sinusoids in the control group. While degenerative hepatocyte and degenerative hepatocyte plaques, mononuclear cell infiltration, sinusoid degeneration, and necrosis were observed to be quite intense in the hypercholesterolemic group, the presence of hepatocyte, endothelium, glycogen, and sinusoids were detected in the group where Atorvastatin, which is used as the reference drug, was administered. When the test samples were examined histopathologically, mononuclear cell infiltration, macro and microvesicles, hepatocyte cell degeneration, sinusoid endothelium, and granuloma formation in Bentonite Group, GBT, and Flaxseed Group. Blood cells in sinusoid, endothelial cells, capsules were seen in GBTF Group and GBTP Group. In the Psyllium Group fibrous tissue, degerative lobular formation, degenerative hepatocyte, and degenerative sinusoids were found to be quite prominent (Figure 5). In this context, it has been determined that histopathological data and biochemical parameters support each other.

## 4 Discussion

Cholesterol and triglyceride, one of the naturally occurring fats in the body, are significant elements in the structure of biological membranes. They are used to both the biosynthesis of steroid hormones, bile acids, vitamin D, and the energy production for the body. However, high cholesterol concentration in the blood is associated with atherosclerosis and increases the risk of cardiovascular diseases. Total cholesterol concentration in the blood affects by both dietary cholesterol content and cholesterol synthesized in the liver (Avcı et al., 2006).

In our study, flaxseed, psyllium, and food-grade bentonite clay did not significantly reduce total cholesterol and LDL-C; however, GBTF Group, GBTP Group, and GBT Group interventions had a significant effect in reducing both total cholesterol levels and LDL-C levels. Therefore, it can be thought that this significant effect is due to the synergistic effect of the raw materials. It was observed that the addition of psyllium to the group with bentonite, grape seed extract, and turmeric extract (GBT Group) provided an 8.6% reduction in total cholesterol. Instead, the addition of flaxseed instead of psyllium to the same group (GBT Group) decreased total cholesterol by 15.9%. According to our results of fixed oil analysis, more amount of oil (better yield) is obtained from flaxseed than psyllium. The difference was found in the ratios of polyunsaturated fatty acids in flaxseed and psyllium, which have relatively the same ratios of saturated and monounsaturated fatty acids in fixed oil analysis. Linolenic Acid/Linoleic Acid (omega-3/omega-6) ratio in flaxseed seems to be more than in psyllium. Therefore, when studied at the same doses with flaxseed and psyllium, the group containing flaxseed (GBTF Group) had further total fatty acids and a higher omega-3/omega-6 ratio than the group containing psyllium (GBTP Group). This situation may explain the different effects on TC and LDL-C changes between these groups (GBTF Group—GBTP Group). In addition, Soltanian and Janghorbani (Soltanian and Janghorbani, 2019) previously stated that flaxseed has superior hypocholesterolemic effects compared to psyllium. Moreover, Brown et al. (1999) state that soluble fibers provide a reduction in serum TC and LDL-C. Similarly, in this study, more reductions in TC and LDL-C levels were observed with the addition of fiber-rich flaxseed or psyllium seeds to the group, which has food-grade bentonite clay, grape seed extract, and turmeric extract (GBT Group).

Low-density lipoprotein (LDL) transports cholesterol from the liver to the tissues, while high-density lipoprotein (HDL) facilitates for transport of cholesterol from peripheral tissues to the liver. Therefore, it is recommended that increase the HDL-C ratio in serum while lowering LDL-C levels to reduce the risk of cardiovascular disease (Nofer et al., 2002). In this study, an increase in HDL-C was found higher in GBTF Group, GBTP Group, GBT Group, and Flaxseed Group than the other groups. Li et al. (2015) mentioned that anthocyanins had an additive effect on HDL-C. Therefore, the rich anthocyanin content of grape seed extract in GBTF Group, GBTP Group, and GBT Group may be responsible for this significant effect in the study. On the other hand, the significant increase in HDL-C only with flaxseed intervention (Flaxseed Group) was inconsistent with the data in the literature because there was no significant increase in HDL-C in previous intervention studies with flaxseed (Shakir and Madhusudhan, 2007).

As a result, considering the total cholesterol, HDL-C, LDL-C, and triglyceride serum concentrations in hypercholesterolemic mice, it was concluded that GBTF Group, GBTP Group, and GBT Group had a positive effect on reducing the risk of cardiovascular diseases such as atherosclerosis.

Hypercholesterolemia causes fatty liver and increases liver enzymes (Choe et al., 2001). Therefore, in this study, various serum parameters were also evaluated to reveal the effects of the test materials on hepatic markers and glucose (Table 4). Previous studies reported that both lipid peroxidation levels increased in serum (Das et al., 2000) and plasma MDA levels (Szczeklik et al., 1985). For this reason, the values measured in Table 4, such as MDA, are essential to evaluate the effects of hypercholesterolemia on the liver. It concluded that GBTF Group and GBTP Group interventions had a significant reducing effect on both MDA and liver enzymes, so these interventions may have positive effects on complications such as fatty liver that may occur as a result of hypercholesterolemia in this study. Moreover, the significant inhibitory effect of flaxseed intervention (Flaxseed Group) on liver enzymes suggests that even flaxseed intervention alone may be sufficient to exhibit liver-protective properties.

A high-fat diet causes lipid accumulation in visceral tissues and increases body weight (Milagro et al., 2006; Yang et al., 2006). The findings obtained due to increased leptin values in the hypercholesterolemic group compared to the control group are consistent with the literature (Table 4). GBTF Group and GBTP Group interventions reduced leptin levels in our study. Similarly, this kind of effect might be due to the synergistic effect of raw material components.

It is an critical issue to examine the correlation between the capacity of antioxidants for scavenging free radicals and that for inhibition of lipid peroxidation (Niki et al., 2008). A significant inhibitory effect on lipid peroxidation was observed in which turmeric extract containing 95% curcumin and grape seed extract rich in antioxidant properties were used (GBTF Group, GBTP Group, and GBT Group) as expected in this study (Table 5).

**TABLE 5:** Effects of test materials on SOD, CAT, LPO, GSH and GPx levels.

*Material*	SOD (μg/mg protein)	CAT (μmol/mg)	LPO (nmol/mg)	GSH (nmol/g)	GPx (U/g Hb)
Control group	2.95 ± 0.51	22.3 ± 4.1	3.12 ± 0.51	1.16 ± 0.51	145.2 ± 5.13
Hypercholesterolomic group	1.57 ± 0.34	10.1 ± 4.0	4.98 ± 0.49	1.02 ± 0.58	102.6 ± 4.73
Bentonite Group	2.04 ± 0.23	19.4 ± 2.7	2.52 ± 0.37	1.18 ± 0.36	145.2 ± 5.13
GBTF Group	5.72 ± 0.13*	48.4 ± 3.1**	1.75 ± 0.13*	2.84 ± 0.09**	229.4 ± 3.48**
GBTP Group	4.39 ± 0.28*	37.4 ± 2.5*	1.86 ± 0.09*	2.51 ± 0.11*	215.1 ± 3.72**
GBT Group	4.46 ± 0.31*	25.8 ± 1.3	1.92 ± 0.27*	1.98 ± 0.22	207.8 ± 4.06*
Flaxseed Group	3.69 ± 0.86	23.5 ± 3.2	2.20 ± 0.41	1.74 ± 0.25	192.3 ± 3.98
Psyllium Group	3.11 ± 0.27	22.6 ± 2.6	2.19 ± 0.36	1.81 ± 0.17	162.2 ± 3.21
*Atorvastatin(10 mg/kg)*	4.37 ± 0.34*	42.3 ± 1.5*	1.43 ± 0.14*	2.37 ± 0.14*	198.7 ± 3.07**

*p < 0.05; **p < 0.01; ***p < 0.001 (significance relative to hypercholesterolemic group).

Bentonite Group: HCD + bentonite clay (60 mg/day); GBTF group: HCD + bentonite clay (60 mg/day) + flaxseed (7 mg/day) + grape seed extract (2.5 mg/day) + turmeric extract (2 mg/day); GBTP group: HCD + bentonite clay (60 mg/day) + psyllium (7 mg/day) + grape seed extract (2.5 mg/day) + turmeric extract (2 mg/day); GBT group: HCD + bentonite clay (60 mg/day) + grape seed extract (2.5 mg/day) + turmeric extract (2 mg/day); Flaxseed Group: HCD + flaxseed (7 mg/day); Psyllium Group: HCD + psyllium (7 mg/day).

Reactive oxygen species (ROS) are reduced by non-enzymatic antioxidant defenses such as ascorbic acid (vitamin C), alpha-tocopherol (vitamin E), and GSH, or by enzymatic antioxidant defenses such as CAT, POD, and SOD (Seet et al., 2010). GSH is also the major non-protein thiol involved in antioxidant cellular defense (Di Pietro et al., 2010). These enzymes were evaluated as effective parameters in detoxification. These enzymes control the number of free radicals produced or scavenge them and prevent their binding to macromolecules. Moreover, the most crucial feature of the antioxidant defense system is that all synergistic components assign against reactive oxygen species for homeostasis. Combined oxidants/antioxidants are more effective than existing alone in the blood (Kaya et al., 2015). Significant increases in SOD, catalase, GSH, and GPx levels were observed in GBTF Group and GBTP Group. Similarly, this kind of effect can be attributed to the presence of curcumin and polyphenols. Also, it can be said that the presence of flaxseed in GBTF Group and psyllium seeds in GBTP Group was responsible for the significant effect on GSH and CAT levels. It is conceivable from other studies that flaxseed oil content and flaxseed lignan content may be responsible for the effects on these parameters. However, more research is necessary on the responsible component in psyllium seed that can be attributed to this kind of effect on these parameters. Contrary to expectations, bentonite did not have a significant effect on some detoxification parameters by itself. However, the short duration of the study can be a limiting factor for this kind of evaluation.

In conclusion, it was shown that GBTF Group, which contains bentonite, flaxseed, grape seed extract, turmeric extract, and GBTP Group, which contains bentonite, psyllium, grape seed extract, and turmeric extract, had significant positive effects on both detoxification parameters and serum cholesterol values when compared to the hypercholesterolemic group. According to a study, bentonite increases fecal lipid excretion by immobilizing lipids in the gastrointestinal system (Xu et al., 2016). The component responsible for the hypocholesterolemic effect of flaxseed is not clear. Nevertheless, we can say that fecal fat excretion was increased with flaxseed fiber (Kristensen et al., 2012). One of the components responsible for the cholesterol-lowering effect in flaxseed is lignans such as secoisolariciresinol diglucoside (SDG). However, the mechanism for the observed cholesterol-lowering effect of flaxseed lignan has not been elucidated (Fukumitsu et al., 2010). The phytoestrogen lignan may be similar to the selective estrogen receptor modulators for the cholesterol-lowering effect (Zhang et al., 2008). Psyllium has a cholesterol-lowering effect by increasing the excretion of bile acids, reducing intestinal cholesterol absorption, and reducing hepatic cholesterol synthesis. Especially high viscosity fibers such as psyllium significantly inhibit cholesterol absorption in the small intestine (Dikeman and Fahey, 2006). Curcumin can regulate the expression of genes involved in cholesterol homeostasis (Srinivasan and Sambaiah, 1991). Besides, It was reported that the anti hyperlipidemia effect of curcumin is similar to that of statins such as lovastatin and that curcumin mainly acts by reducing liver cholesterol biosynthesis by inhibiting HMG-CoA reductase (Zingg et al., 2013). Finally, grape seeds have cholesterol-lowering activity by inhibiting pancreatic cholesterol esterase, binding bile acids, and reducing the solubility of cholesterol in micelles, resulting in delayed cholesterol absorption (Ngamukote et al., 2011). Because of the different cholesterol-lowering mechanisms of all these components, it is hard to make a definitive judgment about the cholesterol-lowering mechanism of action of the combinations. On the other hand, it can be thought that the combinations provide inhibition of cholesterol absorption from the gastrointestinal tract. Studies on the cholesterol-lowering mechanism of these combinations may be conducted in the future. In this study, the model of hypercholesterolemia caused by a high cholesterol diet was studied. Therefore, we can not claim that these combinations would also positively affect a non-dietary hypercholesterolemia model. Also, these findings can be supported by clinical studies. The raw material doses used in the study can be applied to human nutrition, and formulations can be developed in line with this kind of positive effect.

## Data Availability

The original contributions presented in the study are included in the article/Supplementary Material, further inquiries can be directed to the corresponding authors.
